# The impact of COVID-19 pandemic on AMI and stroke mortality in Lombardy: Evidence from the epicenter of the pandemic

**DOI:** 10.1371/journal.pone.0257910

**Published:** 2021-10-01

**Authors:** Camillo Rossi, Paolo Berta, Salvatore Curello, Pietro Giorgio Lovaglio, Mauro Magoni, Marco Metra, Aldo Maria Roccaro, Stefano Verzillo, Giorgio Vittadini

**Affiliations:** 1 ASST Spedali Civili of Brescia, Brescia, Italy; 2 Department of Statistics and Quantitative Methods and CRISP, University of Milan-Bicocca, Milan, Italy; 3 European Commission, Joint Research Centre (JRC), Ispra, Italy; Fondazione IRCCS Policlinico San Matteo, ITALY

## Abstract

**Background:**

The first Covid-19 epidemic outbreak has enormously impacted the delivery of clinical healthcare and hospital management practices in most of the hospitals around the world. In this context, it is important to assess whether the clinical management of non-Covid patients has not been compromised. Among non-Covid cases, patients with Acute Myocardial Infarction (AMI) and stroke need non-deferrable emergency care and are the natural candidates to be studied. Preliminary evidence suggests that the time from onset of symptoms to emergency department (ED) presentation has significantly increased in Covid-19 times as well as the 30-day mortality and in-hospital mortality.

**Methods:**

We check, in a causal inference framework, the causal effect of the hospital’s stress generated by Covid-19 pandemic on in-hospital mortality rates (primary end-point of the study) of AMI and stroke over several time-windows of 15-days around the implementation date of the State of Emergency restrictions for COVID-19 (March, 9^th^ 2020) using two quasi-experimental approaches, regression-discontinuity design (RDD) and difference-in-regression-discontinuity (DRD) designs. Data are drawn from Spedali Civili of Brescia, one of the most hit provinces in Italy by Covid-19 during March and May 2020.

**Findings:**

Despite the potential adverse effects on expected mortality due to a longer time to hospitalization and staff extra-burden generated by the first wave of Covid-19, the AMI and stroke mortality rates are overall not statistically different during the first wave of Covid-19 than before the first peak. The obtained results provided by RDD models are robust also when we account for seasonality and unobserved factors with DRD models.

**Interpretation:**

The non-statistically significant impact on mortality rates for AMI and stroke patients provides evidence of the hospital ability to manage -with the implementation of a dual track organization- the simultaneous delivery of high-quality cares to both Covid and non-Covid patients.

## Introduction

The Covid-19 pandemic has enormously impacted the delivery of clinical healthcare and hospital management practices in most of the hospitals around the world. If on the one hand an extraordinary effort has been exerted to face the workload due to hospital admissions of Covid-19 patients on the other hand it is important to assess whether the clinical management of non-Covid cases has not been compromised during the first unexpected epidemic outbreak.

Around the world, non-Covid hospital admissions felt precipitously with the declaration of the coronavirus (Covid-19) pandemic [1]. Volumes declines for elective surgery or non-critical patients’ medical services, as well as for acute cases like strokes and acute myocardial infarctions ([2–6]) have been registered all over the world.

Declining hospitalization rates may indicate that patients defer care for life-threatening conditions with substantial damage to public health. Moreover, changes in admissions rates may differ by medical condition/diagnosis and ultimately by illness severity which may reflect in significant changes of in-hospital mortality rates.

It can be reasonably stated that if Covid-19 has modified hospitalization selection criteria (cutting hospitalizations for less severe patients), we may expect similar patterns on (raising) in-hospital mortality, unless organizational changes implemented at the hospital level to face the pandemic have ensured appropriate levels of clinical care for patients with non-Covid clinical conditions.

It has been stated that non-Covid patients are not seeking hospital admissions because of their concerns about the risk of nosocomial Covid-19 infection, as well as of the social limitations put in place from governments and local health authorities to face the pandemic ([7, 8]).

In an interesting editorial on the New England Journal of Medicine, Lisa Rosenbaum [9] defines this issue as an “untold toll” asking “*As the coronavirus pandemic focuses medical attention on treating affected patients and protecting others from infection*, *how do we best care for people with non–Covid-related disease*?”. To this aim, monitoring the occurred changes at hospital level for non-Covid cases is crucial for all the National Health Systems (NHS).

To assess this issue, literature has focused on a particular subgroup of hospital admissions. Patients with acute myocardial infarction (AMI) and stroke -needing non-deferrable emergency cares- may be considered the natural candidates as non-Covid patients to be studied.

Recent literature shows how -for urgent diseases- the time from onset of symptoms to the emergency department (ED) presentation has increased in Covid-19 times in Lombardy Region ([10, 11]). In addition, recent evidence from UK show how the pool of admitted patients for AMI was easier to be admitted in hospital (due to a younger and less severe case-mix) and, for NSTEMI and AMI, had higher 30-day mortality during the pandemic ([12, 13]). Similar results were also found in Norther Italy for patients with acute coronary syndrome [13].

Finally, a recent multicenter observational report from Italy found that AMI-related hospitalizations were reduced by almost 50% during the Covid-19 period and accompanied by a threefold increase in mortality and complications [3].

To the best of our knowledge, such papers are mostly observational studies, showing descriptive analysis (correlations, relative risks, or odds ratio) or focusing on mortality trends for non-Covid patients [1], reporting de facto results before and after the Covid period after having established some meaningful cut-off date (first case, lockdown etc.).

However, there is still a lack of such evidences in a causal inference framework using quasi-experimental methods when there is potential endogeneity on comparisons and other factors that could affect comparisons, such as seasonality in the assessed periods [14].

In this end, some US [6] and Italian studies [13] demonstrate that the fall of incidence of hospitalizations for AMI during the first wave of Covid-19 pandemic, declined more than expected by typical seasonal variation alone.

The aim of the present paper is to assess if the extra-burden on hospital’s management and staff (in one hospital in the epicenter of the pandemic in Italy) caused by Covid-19 pandemic has had consequences on intra-hospital mortality for patients admitted for non-Covid urgent conditions. Beside mortality, we also assess if the case-mix of hospitalized patients changed during the epidemic with respect to non-pandemic periods both in short and long-term perspectives. The analysis focused on the first wave of the pandemic only allowing us to observe the very first reaction of the healthcare system to this unexpected event.

Data refer to hospitalizations of patients admitted for stroke and AMI at Spedali Civili, the main hospital in the province of Brescia, one of the most hit provinces in Italy by Covid-19 during the first wave. In fact, as reported by the Italian Statistical Institute, in March 2020 this province experienced an overall mortality increase of 292% compared with the average of the same month in 2015–2019, and by the end of April it had registered 2,500 confirmed Covid-19 deaths [15].

The proposed analysis sheds light on potential effects of organizational and clinical practice changes to face Covid-19 in assuring high quality care for non-deferrable acute admissions in a pandemic period.

To this end, it is important to note that Spedali Civili has implemented specific operational protocols to deliver an appropriate hospital care of non-Covid-19 emergency cases since the beginning of the first Covid-19 wave.

In fact, a progressive increase in Covid-19-devoted beds, either non-ICU or ICU-specific, rapidly reached around 800 beds out of a total of 1547 beds at the end of March 2020 (see Fig A1 in S1 Appendix). To face this situation, ER admissions were structurally modified in a fully ***dual track system*** by introducing a Covid-19-devoted triage and building external emergency tents dedicated to Covid-19 admissions only. Spedali Civili was then literally transformed into a Covid-19 hospital-hub meaning that a drastic modification has been realized both at structural and, most importantly, at organizational level: the already existing staff has been primarily involved in handling the emergency. Physicians, nurses and sanitary workers from infectious diseases wards, ICU, and pneumology received a specific training on Covid-19 management, also internal medicine doctors, cardiologists, neurologists, surgeons, and immunologists together with the related nurses were also active part of Covid-19 patient care delivery.

However, despite the general prioritization of staff and resources on Covid-19 patients, for some time-dependent conditions (such as stroke, cardiovascular emergencies, neurosurgical emergencies, and trauma) an organization based on a “hub-and-spoke” model has been adopted and Spedali Civili was selected as the main regional “hub” in the eastern part of the Lombardy region. Requirements for being selected as “hub” included the “*presence of an integrated trauma team 24/7 on active duty and supplementary surgical teams available on call*, *fast-track access to Emergency Department to reduce interpersonal contact between patients*, *activation of separated pathways to assist and operate on COVID-19 and non-COVID-19 patients*, *and integration of local medical teams with those of the spoke centers*” (see [16] for more details). Within the described dual-track system Ami and Stroke Covid-19-positive patients were admitted following a separated pathway with respect to non-Covid Ami and Stroke patients. Moreover, they received the appropriate treatments generally delivered to patients with AMI and Stroke in addition to some specific cares known to be effective in reducing the Covid infection, as outlined in the literature ([17, 18] and similar to Table 4 of [19]) and recently reported in a guidance document of the The European Society for Cardiology [20].

## Methods

The paper is an observational retrospective, pre- and post-implementation study using registry data from an important hospital set in the epicenter of the Lombardy region, where the Covid-19 epidemic has had a relevant impact in March-May 2020.

The main goal is to investigate the impact of Covid-19 pandemic on patients’ mortality for AMI and stroke acute hospital admissions.

Specifically, the main end-point of the analysis is to study changes of in-hospital mortality rates for AMI and stroke patients.

A standard causal inference method aims at estimating credible causal effects of treatments or policies in a quasi-random framework, when randomized controlled trials (RCTs) are not possible or not ethically/practically feasible.

Quasi-experimental techniques are very useful in this context and may provide a robust tool to draw information on causal impacts. To this end, the regression discontinuity design (RDD, [21]) is a quasi-experimental method that takes advantage of clinical or policy decision rules in which people are differentially assigned to a treatment or intervention if they fall above or below an arbitrary cut-off value of a continuous variable.

Causal inference within an RDD framework comes from the assumption that, aside from differential use of treatment, those on either sides -yet close to the cut-off- are otherwise similar, and validity of the approach relies on the hypothesis that patients on either sides of the threshold have comparable characteristics (as in a pure randomized study).

We take advantage of the sharp cut-off date for the introduction of the Covid-19 lockdown in Italy, which was March 9^th^, 2020, that naturally divides the population of hospitalized patients into a treatment group composed by patients hospitalized after the cut-off date and a control group of patients hospitalized before the cut-off date.

In the RDD analysis patients in the treatment group, those admitted to the Spedali Civili of Brescia hospital with acute stroke or AMI during the first epidemic period (from March, 9^th^ to May, 31^st^ 2020) were compared to patients admitted before the lockdown implementation (January, 1^st^ to March, 8^th^ 2020) for the same diseases.

After having checked for observable differences in the covariates around the cut-off, similarly to [22] we then use calendar week as running variable while the treatment status is identified adopting different time-windows around the cut-off date. In particular, we assessed treatment effect by RDD in several time windows (5 to 10 weeks before and after the cutoff date) assuming that any observed mortality change was due to the hospital-stress introduced by Covid-19 pandemic. We start with a 5 weeks’ time window estimating a Firth’s penalized Maximum Likelihood logistic model [23] to deal with separation and rare events having small sample sizes, which is a relevant issue especially in the shorter time windows around the cut-off date. Having access to regional or national level data would enable us to get a more precise estimate of these effects with shorten time windows around the cut-off date, however at the time of this study the data available only referred to Spedali Civili hospital.

A second analysis was conducted to investigate differences in mortality in an even more robust fashion. Although RDD design addresses the endogeneity of treatment in a quasi-experimental fashion, mortality differences by treatment (in the pandemic period) may be also induced by temporal trends or seasonal factors (i.e. winter vs spring) or by other underlying time-varying factors affecting mortality around the cut-off date. To this end, in the spirit of the difference in difference estimator, we compare mortality in the period surrounding the implementation of the measures in 2020 to the same time period in the two years preceding the Covid-19 pandemic (pooling 2019–18). More explicitly, mortality differences among treated (March, 9^th^ to May, 31^st^ 2020) and control patients (January, 1^st^ to March, 8^th^ 2020) were compared with differences in mortality of patients admitted during the corresponding periods in 2019–18, when no restrictions were in place, and no stress due to the Covid-19 pandemic affected the hospital’s activities.

In this end, we adopt a Difference-in-Regression-Discontinuity design (DRD, see [24] and [22]) which identifies the effect of Covid-19 pandemic on mortality as the difference between the estimated effects on mortality of an RDD around the lockdown date (March, 9^th^) in the year of Covid-19 outbreak (2020) with the ones of an RDD around the same date in the pooled period 2019–18, to control for pre-existing (observed or unobserved) differences in mortality determinants around the cut-off date.

The causal impact was then estimated with a logistic regression that models the probability of death at patient level (outcome variable) as a function of a treatment dummy (assuming value of 1 after the cut-off date and 0 before the cut-off date in 2020), a cohort dummy (equal to 1 for cohort 2020 and 0 for cohorts 2019–18), the running variable (the exposure to treatment, i.e. the week when a patient was evaluated around the cutoff) and their interactions plus other individual covariates to control for unbalanced case-mix among cohorts or among treated and controls in 2020.

The causal effect of DRD is identified as the cohort and treat interaction parameter, whose exponential value can be interpreted as the odds of mortality difference post/pre lockdown in the considered time windows following the implementation of the COVID-19 lockdown (March, 9^th^ 2020) versus the odds of the same event across similar time windows (around the lockdown date) in previous years (2019–18) without the pandemic (See S2 Appendix for methodological details on RDD and DRD models).

However, the choice of the cut-off date may have a different impact on the results of the statistical analyses. Establishing a reliable threshold date is particularly important, especially when we focus on the impact of organizational changes on mortality. Alternative cut-off dates proposed by the literature are February 20^th^ 2020, the date when the first COVID-19 case was identified in Lombardy (Italy) region [13] and February 24^th^ 2020, the date when the first emergency measures were adopted at regional level in Lombardy before the national lockdown date of March 9^th^ ([25, 26]).

To this end, since in a similar context of the same region the reduction of hospitalizations started approximatively two weeks before the beginning of the national lockdown [25], as a robustness check for our estimates, we then implemented a sensitivity analysis according to a new cut-off date artificially set at February 24^th^, 2020 instead of March 9^th^, 2020.

Data are gathered from the hospital information system of Spedali Civili of Brescia and collect anonymized information on all patients admitted from January, 2018 to May, 2020. The electronic hospital discharge data contains basic demographic information (age, gender), information on hospitalization (length of stay, special-care unit use, transfers within the same hospital or through other facilities, and in-hospital mortality), and a total of 6 diagnosis codes and procedures defined according to the International Classification of Diseases, Ninth Revision, Clinical Modification (ICD-9- CM).

The analysis was limited to the following ICD-9- CM codes detected as the principal diagnosis: 410 (Acute Myocardial Infarction) and 434 (Occlusion of cerebral arteries—stroke). We exclude all discharges including as principal diagnosis the code 410.9, corresponding to an AMI with unspecified site. Patients with a principal diagnosis coded 410.7 are detected as AMI with Non-ST Elevation Myocardial Infarction (NSTEMI), while the other 410 codes indicate an AMI with ST Elevation Myocardial Infarction (STEMI).

A set of selected variables at patient level were chosen to control for determinants of patient mortality that may be used in the RDD and DRD models in case of unbalanced case-mix among treated and control groups. In particular, at the patient level we control for patient’s age (in years), gender, foreign status and coexisting conditions identified by the Elixhauser algorithm [27].

To complement the previous analyses, we also examine times from the onset of patients’ symptoms to door and to balloon. In this end, it is important to note that -as pointed out in some recent contributions in the literature- Lombardy region has experienced -on average- an increase in the time from the onset of the first symptoms to the patients hospitalization during the pandemic and we know from the literature how early recognition of symptoms along with shorten time to intervention usually results in better outcomes following both stroke and AMI ([28–30]).

Although times from the onset of patients’ symptoms to door and to balloon are not available for the whole sample of hospitalizations under study, we were able to retrieve aggregated information on a subsample composed by 164 patients hospitalized in the 2020 first lockdown period compared with patients hospitalized in the same period in 2019 years.

## Results

Data refer to 416 hospitalizations in 2020 (224 between March 9^th^–May 31^st^, the post-lockdown period) and 841 (405 in the post-lockdown period), in 2019–18, as reported by totals in Tables 1 and 2. Table 1 illustrates the trend in the number of patients admitted at the hospital with a main diagnosis of AMI (both STEMI and NSTEMI), as well as the trend of patients’ overall mortality rate during the same time periods (Pre and Post lockdown) in each of the last three years.

**Table 1:** Mortality rates of AMI by type (STEMI and NSTEMI) (January 1- May 31, by year).

Year	Stat	STEMI			NSTEMI			TOT		
		*Pre*	*Post*	*Tot*	*Pre*	*Post*	*Tot*	*Pre*	*Post*	*Tot*
2018	Mean (Std. Dev)	0.046 (0.213)	0.108 (0.314)	0.075 (0.265)	0.023 (0.152)	0.044 (0.208)	0.036 (0.188)	0.034 (0.184)	0.067 (0.251)	0.052 (0.223)
	N	43	37	80	43	67	110	86	104	190
2019	Mean (Std. Dev)	0.131 (0.342)	0.074 (0.264)	0.097 (0.298)	0.000 (0.000)	0.037 (0.192)	0.016 (0.128)	0.047 (0.213)	0.056 (0.231)	0.051 (0.222)
	N	38	54	92	67	53	120	105	107	212
2020	Mean (Std. Dev)	0.101 (0.304)	0.048 (0.216)	0.074 (0.263)	0.055 (0.232)	0.000 (0.000)	0.031 (0.175)	0.084 (0.279)	0.033 (0.180)	0.059 (0.237)
	N	59	62	121	36	28	64	95	90	185

**Table 2:** Mortality rates of stroke (January 1- May 31, by year).

Year	Stat	STROKE		
		*Pre*	*Post*	*Tot*
2018	Mean (Std. Dev)	0.011 (0.107)	0.066 (0.249)	0.043 (0.203)
	N	87	121	208
2019	Mean (Std. Dev)	0.094 (0.293)	0.057 (0.234)	0.077 (0.268)
	N	127	104	231
2020	Mean (Std. Dev)	0.041 (0.199)	0.111 (0.316)	0.082 (0.275)
	N	97	134	231

Overall, the number of AMI cases does not change dramatically over time, but we observe that the composition in terms of STEMI and NSTEMI changes in the last year if compared to 2019–18. In 2018 and 2019 STEMI represents the 40% of the AMI admitted at Spedali Civili, while in 2020 the number of STEMI rises to 65% of the total. Suggestive evidence points out how this difference in AMI composition may be driven by modifications in the hospital catchment area with the local healthcare system funneling many STEMI cases from decentralized hospitals (or some smaller cardiovascular and neurological departments which were rapidly closed just after the beginning of the pandemic) to the Spedali Civili, which was the hub hospital in the Brescia province in the considered period.

In addition, we observe an almost steady total mortality rate for patients affected by AMI (around 5% with a non-statistically significant difference of 0.0007, p-value = 0.513) over the first two years considered (2018 and 2019). The overall AMI mortality rate is still non-significantly different in 2020 if compared to 2019 (difference -0.007, p-value = 0.371). These first descriptive comparison points towards a lack of significant change in volumes of total admissions and mortality, suggesting a likely adequate response for this kind of time-depending and high-risk clinical condition, despite the pandemic exposed the hospital to an overwhelmed additional and unforeseen stress.

Table 2 exhibits an increase, even if at the limit of the common significance levels (p-value = 0.066), in mortality rates (increase of 0.039) for stroke cases among 2018 and 2020 with a stable trend in 2020 with respect to 2019 (difference of 0.004, p-value = 0.430).

**Fig 1:**
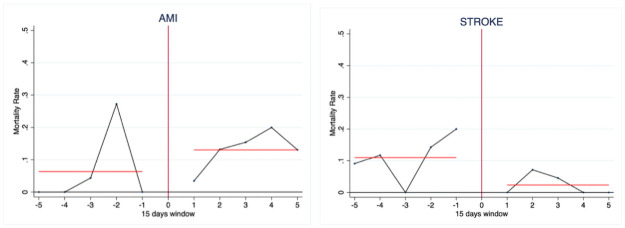
Observed 15-days windows mortality rates around the lockdown cutoff (March, 9^th^ 2020). The red lines identify the average mortality in the pre and post lockdown periods.

In the first analysis, we focused on the comparison of mortality around the national lockdown date (March, 9^th^ 2020) in 2020. Fig 1 reports the observed weekly mortality rates (15-days windows) around the cutoff. It evidences a steady path in mortality after the threshold for AMI patients and a reversed U-shaped pattern for stroke patients.

**Fig 2:**
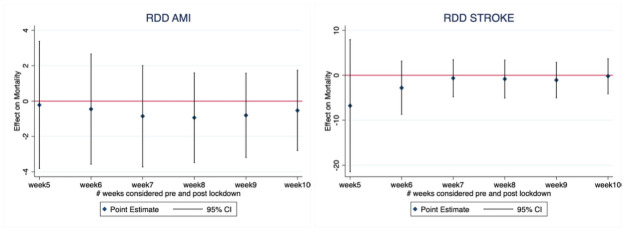
Coefficient (95% CI) indicating mortality differences across various time windows following implementation of the COVID-19 lockdown (March, 9^th^ 2020) versus mortality in similar time windows preceding the measures in 2020. Estimates derived from regression-discontinuity design adopting a Firth logit model.

Fig 2 reports the estimated causal effect of the Covid-19 pandemic around the implementation date of the State of Emergency national restrictions in the RDD model. Results show a non-statistically significant effect of the Covid-19 extra burden on hospitals activities on both AMI and stroke mortality rates in all the 6 different time windows considered around the lockdown date (from 5-weeks to 10 weeks). The RDD Firth’s logistic equation includes also covariates that are not balanced around the cutoff for the largest study period of 10 weeks.

Around this threshold, the two groups are well balanced across the considered covariates except for few case-mix variables and comorbidities, such as length of hospital stay (increased after the cut-off date) and presence of peripheral vascular disorders (whose prevalence decrease after the cut-off) for AMI and diabetes complicated (whose prevalence decreases, although in a not significant manner) for stroke patients (see Tables A1 and A2 in S1 Appendix).

As well as in the RDD case, we check the plausible causal effect of the Covid-19 over several time-windows of 15-days around the implementation date of the State of Emergency restrictions for Covid-19 on March, 9^th^ 2020 when compared with the same mortality difference in the previous years.

Moreover, a comparison among 2020 and 2019–18 cohorts’ characteristics revealed a similar composition of hospitalizations for both AMI and stroke with some exception: AMI patients were slightly younger in 2020 (67.7 vs 70.1), and the quota of NSTEMI significantly decreases in 2020 (33.8% vs 56.2%). Prevalence of comorbidities varies significantly among cohorts for cardiac arrhythmias (increases at 22.5% from 10.1%), peripheral vascular disorders (increases at 5.6% from 1.6%), and other neurological disorders (increased at 1.9% from 0.2%).

As for stroke cases, cohorts are largely balanced for demographics and comorbidities, except for prevalence of patients affected by congestive heart failure (that falls at 0.46% in 2020 from 2.8% in 2018–19). See Tables A3 and A4 in S1 Appendix for more details.

**Fig 3:**
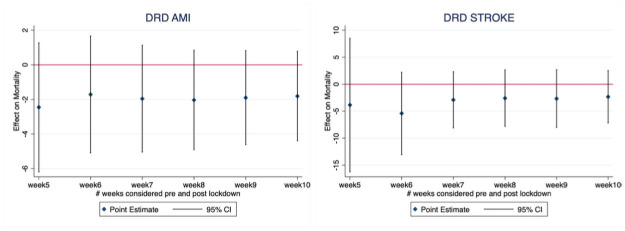
Coefficient (95% CI) indicating mortality difference post/pre lockdown in various time windows following implementation of the COVID-19 lockdown (March, 9^th^ 2020) versus the same event in the similar time windows in previous years (2019–18). Estimates derived from difference in regression-discontinuity design adopting a Firth logit model.

Results of the estimated causal effects on mortality provided by the difference in regression-discontinuity model with the estimates’ confidence intervals (at 95%) are reported in Fig 3. Results of DRD, which controls for unobserved and seasonal effects, confirm the main RDD results: differences are still non-significant and similar in their magnitude at the different time-windows considered, when compared with RDD ones.

For both analyses, differences in mortality are stable over time demonstrating that mortality rates in the Covid-19 period were comparable with pre-Covid-19 levels from the very beginning of the lockdown up to May 2020. Reaction to Covid stress for stroke seemed very well managed since the very beginning with mortality rates in the Covid period non-different from pre-Covid levels up to 10 weeks after the lockdown (end of April).

For what concerns times from the onset of patients’ symptoms to door and to balloon, Table 3 offers descriptive statistics of the mean time (in minutes) occurred from the onset of first symptoms to door and to balloon as well as of the within hospital time between door and balloon. We then registered a statistically significant (at 10% level of confidence only) increase of 2 hours and 51 mins on average from the onset of symptoms to door and a non-significantly different from 2019 but larger time from door to balloon of 18 mins -which may be due to Covid-19 testing procedures at the hospital admission stage- in 2020.

**Table 3:** Descriptive statistics of AMI time from onset symptoms, by year and difference.

Year	N. Obs	Onset Symptoms to Door		Door to Balloon		Onset Symptoms to Balloon	
		Mean (minutes)	Std. Dev.	Mean (minutes)	Std. Dev.	Mean (minutes)	Std. Dev.
2019	64	206	30.13	77.68	11.3	254.72	28.45
2020	100	377.12	85.41	95.82	11.11	476.31	88.7
2019–2020		-171.12	90.57	-18.13	15.86	-221.58	
t-stat		-1.88		-1.14		-2.37	
p-value		0.061		0.254		0.019	

On average a total increase of the global time from the onset of symptoms to Balloon is around 3 hours and 41 mins (statistically significant at a 5% confidence level).

A similar pattern has been experienced also for stroke episodes with an average increase of 10 mins -from 126 mins in 2019 to 136 mins in the first half of 2020- in the onset of symptoms to door time computed using all direct in-hospital admissions via ambulance.

## Discussion

During the first wave of the Covid-19 epidemic, hospitals in many parts of the world were required to operate in crisis capacity and have suffered dramatic reductions of their daily activity, such as urgent surgery and oncological programs ([31, 32]), acute myocardial infarctions treatments [3], acute coronary syndrome admissions [13], pediatric emergency departments activities [25], Emergency Department workload [26], mental health therapies [33] and stroke interventions [5].

Recent studies reported a three-fold increase in AMI-related mortality and complications in Italy during the COVID-19 period if compared with the same period of the year before [3] as well as a significant increase of overall mortality for all causes (+7.5%) -largely due to out-of-hospital mortality (+43.2%) especially related to cardiovascular (+32.7%) diseases [26].

Thus, we expected an increase of in-hospital mortality and complications for patients with non-Covid clinical conditions (AMI and stroke) that require appropriate levels of clinical care in the main hospital of the Brescia province, one of the most hit provinces in Italy by Covid-19. In this context, quasi-experimental methods reveal to be useful tools to get clear causal evidence to support policy makers in pandemic times.

Both RDD and DRD results demonstrates that, although we expected illness severity in non-Covid-19 admissions to increase (due to possible selection criteria that could have skimmed less severe patients) in-hospital mortality in acute non-Covid patients was not different during the first wave of the pandemic with respect both to the pre-lockdown period and previous years. In-hospital mortality rates in the Covid-19 period remain stable with respect to pre-lockdown for both AMI and stroke patients.

These results seem to be even more important in the Italian context where it has been recently found a substantial excess of mortality not accounted in Covid-related deaths, as large as 90% in some municipality during the first peak of the outbreak [34], raising the question of whether some patients died without medical attention during the Covid-19 pandemic [13], especially for outpatients [26].

Concerning admission criteria of hospitalization in the two analyzed periods (2020 vs 2019–18), patients’ characteristics of hospitalized patients are largely similar, with some exceptions (Cardiac Arrhythmias and Peripheral Vascular Disorders, which are significantly increased in 2020 for AMI patients, unlikely a large reduction in NSTEMI patient). A similar decreasing pattern was observed for Congestive Heart Failure patients admitted for stroke.

Moreover, the key finding of our study, i.e. a not significant increase of Spedali Civili in-hospital mortality for cardiovascular and stroke patients during lockdown, differs from findings of other Italian studies ([13, 26, 35]) pointing out the crucial role of appropriate hospital management strategies to prevent adverse effects on non-Covid acute cases.

In fact, results show that non-Covid cases (in particular, non-deferrable cases) can be effectively managed adopting a fully dual track system in the hospital setting which helps in avoiding that the unexpected extra-stress due to Covid-19 hospitalizations would compromise the quality of cares to provided.

Another relevant point in the analysis is the choice of the cut-off date: a different date may have a different impact on the results of the statistical analyses. In a recent Italian study, comparisons using different threshold date (i.e. February, 24^th^) found no significant differences in overall and in-hospital mortality with respect to one year before the pandemic [26]. Our sensitivity analyses, which use the same alternative cut-off date, confirms these findings as well as our main results.

However, March 9^th^ seems to us the appropriate reference date, given that our study aims at assessing potential differences in AMI and stroke mortality rates due to the extra hospital stress induced by the admission of a relevant number of Covid-19 patients. This credibly started to affect hospitals management and daily organization after March 9^th^, 2020 and not in the two weeks before (see Fig A1 of S1 Appendix).

Finally, we observed an increase in average times to door and time to balloon after the lockdown.

Our estimates (onset of symptoms to Balloon) is in line with recent studies regarding Humanitas hospital in Lombardy [30].

While we directly cannot assess out-of hospital mortality with the available data (which we likely suppose increased a lot given the large increase in time from the onset of symptoms to door), we are able to provide evidence on in-hospital mortality. We thus expect an in-hospital mortality rate significantly different compared to the previous years. However, our RDD and DRD results show that there wasn’t any difference between the treated and control groups. One explanation of the non-significant difference in mortality between the great 2020 lockdown period and the same period in 2019–18 may be the results of a good hospital management of AMI and stroke cases given the implemented dual-track organizations. More explicitly, having a dedicated admission to the Emergency Department and a 24/7 dedicated team of doctor and nurses as well as ICU beds availability, it seems to have contributed in containing the potential negative effects of longer times to hospitalization, at least for what concerns the hospital possibilities.

An alternative explanation may relate the insignificant difference to the result of the disease itself, which does not necessarily show an increase in mortality rates when below a certain value of the door to balloon time. For example, for AMI patients this value is equal to a 95 mins door to balloon time [36] similar to the one registered at Spedali Civili in 2020, which may partially explain our results. In fact, the increase in onset to door time for AMI patients remains particularly relevant, seriously harming patients’ probability to survive before being hospitalized. In addition, the ability of keeping the time from door to balloon below 95 mins for AMI should be attributed either to good management practices or to pure luck (e.g. the time to perform a Covid-19 test is short enough not to harm the total door to balloon time). Which of the two explanations is true still remains an open question for which our analysis cannot offer an exhaustive response. Similar reasoning applies for stroke cases.

At least four limitations need to be considered this study. First, the proposed evidence is limited to one single hospital, and this might not reflect the clinical outcomes at every hospital in Northern Italy during the first wave of the pandemic. Availability of similar data for the national or regional context would be helpful in further substantiate the obtained findings and in exploring existing differences in efficacy of different managerial settings implemented in Lombardy hospitals.

Second, we only measure in-hospital mortality and therefore we are unable to quantify neither mortality for not hospitalized patients or mortality post-discharge (i.e. 30 days mortality), potentially underestimating the overall true mortality rate. Considering the relevant increase in time from the onset of symptoms to hospital presentation, indeed data on other outcomes also need to be considered (e.g. in-hospital heart failure, need for inotropic infusion, ejection fraction at discharge) in future studies to investigate more in depth if any relevant adverse effect (in addition to mortality rate) has been experienced by AMI and stroke patients during Covid-19 time [37, 38].

Moreover, we think it is particularly important to study which management practices at hospital level revealed to be effective in containing the potential adverse effects of the Covid-19 hospital stress, specifically for non-Covid patients being hospitalized during the first wave. Third, our analysis reflects less than a full year of Covid-19 data ending in May 2020. Fourth, unfortunately, it is not possible to formally consider the time-to-hospitalization as a covariate in our models given the availability of this information only at aggregated level for stroke cases and for a subsample of AMI patients.

## Conclusions

To our knowledge, our study is the first attempt to assess the impact of COVID-19 and related burden and hospital stress on in-hospital mortality for AMI and stroke in one of the largest Northern Italian hospitals sited in the province of Brescia (the pandemic epicenter in Europe in the first wave, recording 2,500 confirmed COVID-19 deaths by the end of April 2020) in a causal inference framework being able to control for seasonal and other unobserved factors that were possibly in place before the pandemic.

Results agree that -even if the extra-burden faced during the lockdown period has changed many of the ordinary activities of the hospital- acute AMI and stroke mortality have not been affected by Covid-19 stress experienced by the healthcare system, demonstrating that the mitigation strategies put in place by the Spedali Civili of Brescia to simultaneously face Covid-19 and non-Covid-19 healthcare delivery have been successfully implemented.

This study suggests that effective organizational changes (i.e. a dual track system) are able to face the adverse effects of the pandemic in terms of in-hospital mortality containment for AMI and stroke patients, ensuring appropriate levels of clinical care for admitted patients with non-Covid and non-deferrable clinical conditions. Moreover, as suggested by recent studies [38], better communication strategies should be implemented in epidemic times by public health authorities to ensure timely presentation of individuals (preventing the huge increase in time to door registered during Covid-19 outbreak) with acute non-deferrable diseases such as AMI and stroke.

This is particularly important in a regional context where the strong impact caused by Covid epidemic has moved experts and health managers on re-thinking the system supporting the best practices which have been able to timely and safely help in reacting to the unexpected health emergency ([39, 40]).

## Supporting information

S1 Appendix Beds occupancy, patients characteristics and sensitivity analysis. (DOCX)

S2 Appendix Statistical methods [41]. (DOCX)

## References

[1] Birkmeyer JD, Barnato A, Birkmeyer N, et al. The Impact of the COVID-19 pandemic on hospital admissions in the United States. Health Affairs 2020 39:11, 2010–2017. doi: 10.1377/hlthaff.2020.00980 32970495 PMC7769002

[2] Arcaya MC, Tucker-Seeley RD, Kim R, et al. Research on neighborhood effects on health in the United States: a systematic review of study characteristics. Soc Sci Med. 2016; 168:16–29. doi: 10.1016/j.socscimed.2016.08.047 27637089 PMC5065354

[3] De Rosa S, Spaccarotella C, Basso C, et al. Reduction of hospitalizations for myocardial infarction in Italy in the COVID-19 era. Eur Heart J. 2020; 41(22): 2083–2088. doi: 10.1093/eurheartj/ehaa409 32412631 PMC7239145

[4] Mahmud N, Hubbard RA, Kaplan DE, Serper M. Declining cirrhosis hospitalizations in the wake of the COVID-19 pandemic: a national cohort study. Gastroenterology. 2020; 159(3):1134–1136.e3. Crossref, Medline, Google Scholar. doi: 10.1053/j.gastro.2020.05.005 32387493 PMC7200325

[5] Siegler JE, Heslin ME, Thau L, et al. Falling stroke rates during COVID-19 pandemic at a comprehensive stroke center. J Stroke Cerebrovasc Dis. 2020; 29(8):104953. Crossref, Medline, Google Scholar. doi: 10.1016/j.jstrokecerebrovasdis.2020.104953 32689621 PMC7221408

[6] Solomon MD, McNulty EJ, Rana JS, et al. The COVID-19 pandemic and the incidence of acute myocardial infarction. N Engl J Med. 2020; 383(7):691–693. doi: 10.1056/NEJMc2015630 32427432

[7] Abdelaziz HK, Abdelrahman A, Nabi A, et al. Impact of COVID-19 pandemic on patients with ST-segment elevation myocardial infarction: insights from a British cardiac center. Am Heart J. 2020; 226:45–48. doi: 10.1016/j.ahj.2020.04.022 32497914 PMC7211651

[8] Huet F, Prieur C, Schurtz G, et al. One train may hide another: acute cardiovascular diseases could be neglected because of the COVID-19 pandemic. Arch Cardiovasc Dis. 2020; 113:303–307. doi: 10.1016/j.acvd.2020.04.002 32362433 PMC7186196

[9] Rosenbaum L. (2020). The untold toll—the pandemic’s effects on patients without covid-19. N Engl J Med. 2020; 382(24), 2368–2371 doi: 10.1056/NEJMms2009984 32302076

[10] Gramegna M, Baldetti L, Beneduce A, et al. ST-Segment–Elevation myocardial infarction during COVID-19 pandemic: insights from a regional public service healthcare hub. Circ Cardiovasc Interv. 2020 Aug;13(8):e009413. doi: 10.1161/CIRCINTERVENTIONS.120.009413 32791953

[11] Mitra B, Mitchell RD, Cloud GC et al. Presentations of stroke and acute myocardial infarction in the first 28 days following the introduction of state of emergency restrictions for COVID-19. Emerg Med Australas. 2020; 32(6):1040–1045. doi: 10.1111/1742-6723.13621 32833297 PMC7461453

[12] Rudilosso S, Laredo C, Vera V et al. Acute stroke care is at risk in the era of COVID-19: experience at a comprehensive stroke center in Barcelona. Stroke. 2020; 51(7):1991–1995. doi: 10.1161/STROKEAHA.120.030329 32438895 PMC7258755

[13] De Filippo O, D’Ascenzo F, Angelini F, et al. Reduced rate of hospital admissions for ACS during Covid-19 outbreak in northern Italy. N Engl J Med 2020; 383: 88–89. doi: 10.1056/NEJMc2009166 32343497 PMC7224608

[14] Sipilä JO, Ruuskanen JO, Kauko T, et al. Seasonality of stroke in Finland. Ann Med. 2017; 49(4):310–318. doi: 10.1080/07853890.2016.1254350 27786555

[15] Istat (2020) Impact of the Covid-19 epidemic on the total mortality of the resident population in the first quarter of 2020. Rome: Istat press.

[16] Casiraghi A, Domenicucci M, Cattaneo S, et al. Operational strategies of a trauma hub in early coronavirus disease 2019 pandemic. Int Orthop. 2020;44(8):1511–1518. doi: 10.1007/s00264-020-04635-5 32506141 PMC7275124

[17] Bikdeli B, Madhavan MV, Jimenez D, Chuich T, Dreyfus I, Driggin E, et al; Global COVID-19 Thrombosis Collaborative Group, Endorsed by the ISTH, NATF, ESVM, and the IUA, Supported by the ESC Working Group on Pulmonary Circulation and Right Ventricular Function. COVID-19 and Thrombotic or Thromboembolic Disease: Implications for Prevention, Antithrombotic Therapy, and Follow-Up: JACC State-of-the-Art Review. J Am Coll Cardiol. 2020 Jun 16;75(23):2950–2973. doi: 10.1016/j.jacc.2020.04.031 Epub 2020 Apr 17. . 32311448 PMC7164881

[18] Anker SD, Butler J, Khan MS, Abraham WT, Bauersachs J, Bocchi E, et al. Conducting clinical trials in heart failure during (and after) the COVID-19 pandemic: an Expert Consensus Position Paper from the Heart Failure Association (HFA) of the European Society of Cardiology (ESC). Eur Heart J. 2020 Jun 7;41(22):2109–2117. doi: 10.1093/eurheartj/ehaa461 Erratum in: Eur Heart J. 2021 May 7;42(18):1810. . 32498081 PMC7314099

[19] Paris Sara, Inciardi Riccardo M, Lombardi Carlo Mario, Tomasoni Daniela, Ameri Pietro, Carubelli Valentina, et al Implications of atrial fibrillation on the clinical course and outcomes of hospitalized COVID-19 patients: results of the Cardio-COVID-Italy multicentre study, EP Europace, 2021;, euab146, doi: 10.1093/europace/euab146 34297833 PMC8344555

[20] The European Society for Cardiology. ESC Guidance for the Diagnosis and Management of CV Disease during the COVID-19 Pandemic. https://www.escardio.org/Education/COVID-19-and-Cardiology/ESC-COVID-19-Guidance. (Last update: 10 June 2020)

[21] Hahn J, Todd P, Van der Klaauw W. Identification and estimation of treatment effects with a regression-discontinuity design. Econometrica 2001; 69(1): 201–209.

[22] Been JV, Ochoa LB, Bertens LC, et al. Impact of COVID-19 mitigation measures on the incidence of preterm birth: a national quasi-experimental study. Lancet Public Health 2020; 5(11), e604–e611. doi: 10.1016/S2468-2667(20)30223-1 33065022 PMC7553867

[23] Firth D. Bias reduction of maximum likelihood estimates. Biometrika 1993;80:27–38.

[24] Hong K, Dragan K, Glied S. Seeing and hearing: the impacts of New York City’s universal prekindergarten program on the health of low-income children. NBER Working 2017; Paper No. 23297. 10.1016/j.jhealeco.2019.01.004 30826551

[25] Clavenna A, Nardelli S, Sala D, et al. Impact of COVID-19 on the pattern of access to a pediatric emergency department in the Lombardy region, Italy. Pediatr Emerg Care. 2020;36(10):e597–e598. doi: 10.1097/PEC.0000000000002232 . 32826641

[26] Santi L, Golinelli D, Tampieri A, et al. Non-COVID-19 patients in times of pandemic: Emergency department visits, hospitalizations and cause-specific mortality in Northern Italy. PLoS ONE 2021; 16(3): e0248995. doi: 10.1371/journal.pone.0248995 33750990 PMC7984614

[27] Elixhauser A., Steiner C., Harris, et al. Comorbidity measures for use with administrative data. Med Care. 1998; 36(1):8–27. doi: 10.1097/00005650-199801000-00004 . 9431328

[28] Levine GN, Bates ER, Blankenship JC, et al. 2015 ACC/AHA/SCAI focused update on primary percutaneous coronary intervention for patients with ST-elevation myocardial Infarction: An update of the 2011 ACCF/AHA/SCAI guideline for percutaneous coronary intervention and the 2013 ACCF/AHA guideline for the management of ST-elevation myocardial infarction: A report of the American College of Cardiology/American Heart Association Task Force on Clinical Practice Guidelines and the Society for Cardiovascular Angiography and Interventions. Catheter Cardiovasc Interv. 2016; 87(6):1001–1019. doi: 10.1002/ccd.26325 26489034

[29] Lees KR, Bluhmki E, von Kummer R et al. Time to treatment with intravenous alteplase and outcome in stroke: an updated pooled analysis of ECASS, ATLANTIS, NINDS, and EPITHET trials. Lancet 2010; 375:1695–1703. doi: 10.1016/S0140-6736(10)60491-6 20472172

[30] Asteggiano F, Divenuto I, Ajello D, et al. Stroke management during the Covid-19 outbreak: challenge and results of a hub-center in Lombardy, Italy. Neuroradiology 2021; doi: 10.1007/s00234-020-02617-3 33410950 PMC7788174

[31] Rausei S, Ferrara F, Zurleni T, et al. For Italian association of hospital surgeons dramatic decrease of surgical emergencies during COVID-19 outbreak. J. Trauma Acute Care Surg 2020; 89: 1085–1091. 32890343 10.1097/TA.0000000000002923 PMC7687876

[32] Torzilli G, Viganò L, Montorsi M, et al. On behalf of COVID-SURGE-ITA group. A snapshot of elective oncological surgery in Italy during COVID-19 emergency: pearls, pitfalls, and perspectives. Ann. Surg. 2020; 272:e112–e117. doi: 10.1097/SLA.0000000000004081 32675512 PMC7373476

[33] Holmes EA, O’Connor RC, Perry VH, et al. Multidisciplinary research priorities for the COVID-19 pandemic: a call for action for mental health science. Lancet Psychiatry 2020; 7:547–560. doi: 10.1016/S2215-0366(20)30168-1 32304649 PMC7159850

[34] Blangiardo M, Cameletti M, Pirani M, et al. Estimating weekly excess mortality at sub-national level in Italy during the COVID-19 pandemic. PLoS ONE 2020; 15(10), 1–15. doi: 10.1371/journal.pone.0240286 33035253 PMC7546500

[35] Baldi E, Sechi GM, Mare C, et al. Out-of-hospital cardiac arrest during the Covid-19 outbreak in Italy. N Engl J Med. 2020; 383(5):496–498. doi: 10.1056/NEJMc2010418 32348640 PMC7204428

[36] Menees DS, Peterson ED, Wang Y, et al. Door-to-balloon time and mortality among patients undergoing primary PCI. N Engl J Med. 2013; 369(10):901–909. doi: 10.1056/NEJMoa1208200 . 24004117

[37] Tomasoni D, Adamo M, Italia L, Branca L, Chizzola G, Fiorina C, et al. Impact of COVID-2019 outbreak on prevalence, clinical presentation and outcomes of ST-elevation myocardial infarction. J Cardiovasc Med (Hagerstown). 2020 Nov;21(11):874–881. doi: 10.2459/JCM.0000000000001098 32941325

[38] Zorzi A, Vio R, Rivezzi F. et al. Characteristics and hospital course of patients admitted for acute cardiovascular diseases during the coronavirus disease-19 outbreak. J Cardiovasc. Med. 2021: 22:29–35. doi: 10.2459/JCM.0000000000001129 33186239

[39] Nacoti M, Ciocca A, Giupponi A, et al. At the epicenter of the Covid- 19 pandemic and humanitarian crises in Italy: changing perspectives on preparation and mitigation. NEJM Catal. 2020.

[40] Dunlop C, Howe A, Allen LN. The coronavirus outbreak: the central role of primary care in emergency preparedness and response. BJGP Open 2020; 1;4(1):bigopen20X101041. doi: 10.3399/bjgpopen20X101041 31992543 PMC7330191

[41] Vittinghoff E, Glidden DV, Shiboski SC, et al. Regression Methods in Biostatistics. New York: Springer 2012

