# Supplementary material for: The impact of COVID-19 pandemic on AMI and stroke mortality in Lombardy: Evidence from the epicenter of the pandemic
Source: PLoS One. 2021 Oct 1;16(10):e0257910. doi: 10.1371/journal.pone.0257910 (PMC8486095; doi:10.1371/journal.pone.0257910)
Supplement: S1 Appendix — (DOCX) [file pone.0257910.s001.docx]

**S1 Appendix: Beds occupancy, patients characteristics and sensitivity analysis**

Figure A1: Descriptive statistics of Covid-19 and non-Covid-19 occupied beds in ordinary (Panel A) and acute/ICU wards (panel B) during the first pandemic wave

Table A1: Differences of AMI patients characteristics pre and post lockdown (March, 9^th^) in 2020

| Variables | Pre | Post | p value |
| --- | --- | --- | --- |
| Age (years) | 66.214 | 68.065 | 0.587 |
| Female (%) | 21.4% | 27.1% | 0.648 |
| NSTEMI (%) | 42.9% | 34.2% | 0.518 |
| Length of stay (days) | 4.929 | 7.658 | **0.021** |
| Foreign (%) | 2.14% | 6.5% | 0.044 |
| Congestive Heart Failure (%) | 0.0% | 9.7% | 0.225 |
| Cardiac Arrhythmias (%) | 7.1% | 2.3% | 0.179 |
| Valvular Disease (%) | 14.3% | 5.8% | 0.221 |
| Pulmonary Circulation Disorders (%) | 0.00% | 1.9% | 0.602 |
| **Peripheral Vascular Disorders** (%) | **35.7%** | **5.8%** | **0.000** |
| Hypertension, Uncomplicated (%) | 0.00% | 0.6% | 0.765 |
| Other Neurological Disorders (%) | 0.00% | 1.9% | 0.602 |
| Diabetes, Uncomplicated (%) | 0.00% | 2.6% | 0.546 |
| Hypothyroidism (%) | 0.00% | 0.6% | 0.765 |
| Renal Failure (%) | 0.00% | 3.2% | 0.498 |
| Solid Tumor Without Metastasis (%) | 0.00% | 0.6% | 0.765 |
| Coagulopathy (%) | 0.00% | 0.6% | 0.765 |
| Obesity (%) | 0.00% | 0.6% | 0.765 |
| Fluid and Electrolyte Disorders (%) | 0.00% | 0.6% | 0.765 |
| Blood Loss Anemia (%) | 0.00% | 1.3% | 0.671 |
| Deficiency Anemia (%) | 0.00% | 1.3% | 0.671 |
| Hypertension, Complicated (%) | 0.00% | 1.9% | 0.602 |

Table A2: Differences of stroke patients characteristics pre and post lockdown (March, 9^th^) in 2020

| Variables | Pre | Post | p value |
| --- | --- | --- | --- |
| Age (years) | 67.286 | 72.124 | 0.327 |
| **Female** (%) | **0.00%** | **45.2%** | **0.018** |
| Length of stay (days) | 5.000 | 8.490 | 0.136 |
| Foreign (%) | 14.3% | 6.7% | 0.437 |
| Congestive Heart Failure (%) | 0.00% | 0.5% | 0.856 |
| Cardiac Arrhythmias (%) | 14.3% | 20.5% | 0.690 |
| Valvular Disease (%) | 0.00% | 1.0% | 0.796 |
| Peripheral Vascular Disorders (%) | 0.00% | 2.9% | 0.652 |
| Hypertension, Uncomplicated (%) | 28.6% | 16.7% | 0.412 |
| Other Neurological Disorders (%) | 0.00% | 2.4% | 0.681 |
| Diabetes, Uncomplicated (%) | 0.00% | 4.8% | 0.557 |
| **Diabetes, Complicated** (%) | **14.3%** | **2.4%** | **0.059** |
| Hypothyroidism (%) | 0.00% | 0.5% | 0.856 |
| Renal Failure (%) | 0.00% | 0.5% | 0.856 |
| Lymphoma (%) | 0.00% | 0.5% | 0.856 |
| Solid Tumor Without Metastasis (%) | 0.00% | 0.5% | 0.856 |
| Rheumatoid Arthritis/Collagen Vascular (%) | 0.00% | 0.5% | 0.856 |
| Coagulopathy (%) | 0.00% | 0.5% | 0.856 |
| Fluid and Electrolyte Disorders (%) | 0.00% | 1.9% | 0.714 |
| Blood Loss Anemia (%) | 0.00% | 0.5% | 0.856 |
| Hypertension, Complicated (%) | 0.00% | 1.0% | 0.796 |

Table A3: Differences of AMI patients characteristics between 2020 and 2018/19

| **Variable** | **2018-19** | **2020** | **p value** |
| --- | --- | --- | --- |
| **Age** (years) | **70.089** | **67.763** | **0.049** |
| Female (%) | 30.4% | 26.9% | 0.399 |
| **Nstemi** (%) | **56.2%** | **33.8%** | **0.000** |
| Foreign patient (%) | 6.8% | 6.3% | 0.815 |
| Congestive Heart Failure (%) | 11.2% | 10.0% | 0.668 |
| **Cardiac Arrhythmias** (%) | **10.1%** | **22.5%** | **0.000** |
| Valvular Disease (%) | 3.8% | 5.6% | 0.317 |
| Pulmonary Circulation Disorders (%) | 0.7% | 1.9% | 0.209 |
| **Peripheral Vascular Disorders** (%) | **1.6%** | **5.6%** | **0.008** |
| Hypertension, Uncomplicated (%) | 1.4% | 0.6% | 0.439 |
| **Other Neurological Disorders** (%) | **0.2%** | **1.9%** | **0.031** |
| Chronic Pulmonary Disease (%) | 0.9% | 0.00% | 0.220 |
| Diabetes, Uncomplicated (%) | 1.4% | 2.5% | 0.362 |
| Diabetes, Complicated (%) | 0.2% | 0.00% | 0.541 |
| Hypothyroidism (%) | 0.00% | 0.6% | 0.102 |
| Renal Failure (%) | 2.8% | 3.1% | 0.840 |
| Liver Disease (%) | 0.2% | 0.00% | 0.541 |
| Lymphoma (%) | 0.2% | 0.00% | 0.541 |
| Solid Tumor Without Metastasis (%) | 0.5% | 0.6% | 0.813 |
| Coagulopathy (%) | 0.2% | 0.6% | 0.470 |
| Obesity (%) | 0.2% | 0.6% | 0.470 |
| Fluid and Electrolyte Disorders (%) | 0.2% | 0.6% | 0.470 |
| Blood Loss Anemia (%) | 1.2% | 1.3% | 0.938 |
| Deficiency Anemia (%) | 1.2% | 1.3% | 0.938 |
| Hypertension, Complicated (%) | 0.5% | 1.9% | 0.099 |

Table A4: Differences of stroke patients characteristics between 2020 and 2018/19

| **Variable** | **2018-19** | **2020** | **p value** |
| --- | --- | --- | --- |
| Age (years) | 70.37 | 72.20 | 0.119 |
| Female (%) | 44.9% | 45.1% | 0.959 |
| Foreign patient (%) | 5.5% | 6.6% | 0.571 |
| Congestive Heart Failure (%) | 2.8% | 0.5% | 0.045 |
| Cardiac Arrhythmias (%) | 17.3% | 21.1% | 0.234 |
| Valvular Disease (%) | 3.3% | 0.9% | 0.073 |
| Pulmonary Circulation Disorders (%) | 0.7% | 0.00% | 0.237 |
| Peripheral Vascular Disorders (%) | 4.4% | 2.8% | 0.331 |
| Hypertension, Uncomplicated (%) | 19.3% | 16.9% | 0.466 |
| Paralysis (%) | 0.4% | 0.00% | 0.334 |
| Other Neurological Disorders (%) | 2.4% | 2.4% | 0.963 |
| Chronic Pulmonary Disease (%) | 0.7% | 0.00% | 0.237 |
| Diabetes, Uncomplicated (%) | 6.6% | 4.7% | 0.342 |
| Diabetes, Complicated (%) | 2.0% | 2.8% | 0.491 |
| Hypothyroidism (%) | 0.4% | 0.5% | 0.954 |
| Renal Failure (%) | 1.8% | 0.5% | 0.180 |
| Liver Disease (%) | 0.2% | 0.00% | 0.495 |
| Lymphoma (%) | 0.2% | 0.5% | 0.580 |
| Solid Tumor Without Metastasis (%) | 1.1% | 0.5% | 0.425 |
| Rheumatoid Arthritis (%) | 0.7% | 0.5% | 0.770 |
| Coagulopathy (%) | 0.7% | 0.5% | 0.770 |
| Obesity (%) | 0.2% | 0.00% | 0.495 |
| Fluid and Electrolyte Disorders (%) | 3.3% | 1.9% | 0.309 |
| Blood Loss Anemia (%) | 0.2% | 0.5% | 0.580 |
| Deficiency Anemia (%) | 0.7% | 0.00% | 0.237 |
| Psychoses (%) | 0.2% | 0.00% | 0.495 |
| Depression (%) | 0.2% | 0.00% | 0.495 |
| Hypertension, Complicated (%) | 3.1% | 0.9% | 0.094 |

**Sensitivity Analysis using February, 24^th^ 2020 as cut-off**

**
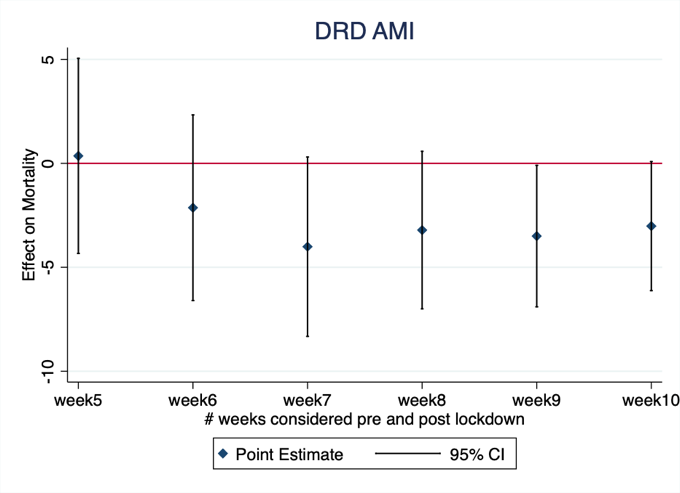

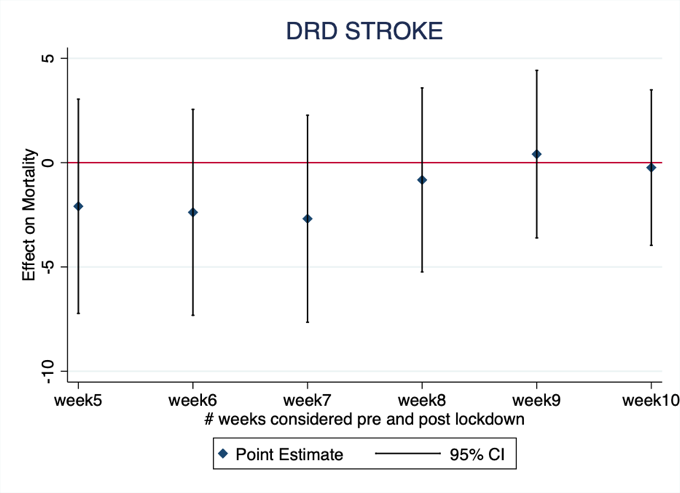
**

Figure A2: coefficient (95% CI) indicating mortality difference post/pre lockdown in various time windows following the first Covid-19 case in Lombardy (February, 24^th^ 2020) versus the same event in the similar time windows in previous years (2019-18). Estimates derived from difference in regression-discontinuity design adopting a Firth logit model.
