# Supplementary material for: The impact of COVID-19 pandemic on AMI and stroke mortality in Lombardy: Evidence from the epicenter of the pandemic
Source: PLoS One. 2021 Oct 1;16(10):e0257910. doi: 10.1371/journal.pone.0257910 (PMC8486095; doi:10.1371/journal.pone.0257910)
Supplement: S2 Appendix — (DOCX) [file pone.0257910.s002.docx]

**S2 Appendix: Statistical Methods**

Let Y_it_ be the outcome variable (Y_it_=1 if subject i is died at time t, 0 otherwise), X_it_ denote the time t observed for patient i (the assignment/running variable that decides the treatment D), and Ι(X_it_ > c) = D_it_ an indicator of eligibility for patients in the study period (year 2020), which equals 1 for patients evaluated at time t after the lockdown fixed at cutoff c in 2020 (treated in the study period), and zero for patients before lockdown in 2020 (control group in the study period).

A Regression discontinuity design (RDD) estimates the effect of lockdown just comparing differences of the outcome variable among treated and controls at the cutoff c, for observations in a small window close to the cutoff c, allowing for different functions to the left (X_it_ - c) and the right (D_it_ = Ι(X_it_ > c)) of the cutoff. Specifying linear functions, we estimate the RDD in the following logistic equation (using Firth penalized maximum likelihood, estimation method):

log(P(Y_it_=1)/P(Y_it_=0)) = α + βD_it_ + γ_1_(X_it_ - c) + γ_2_D_it_(X_it_ - c)

This RDD was estimated within several time-windows around the lockdown date (March 9^th^). Letting h {\displaystyle h} h be the bandwidth of data used on either side of the cutoff, we have c - h < c − h ≤ X ≤ c + h {\displaystyle c-h\leq X\leq c+h} X_it_ < c + h different windows for estimation and different coefficients that fit data.

Once estimated and exponentiated, coefficients can be interpreted as odds ratio: in particular, exp(β) is the ratio of the odds of death after lockdown and the odds of death before the lockdown at the cutoff date for different time windows, thus the treatment/lockdown effect in the study period. Point estimates and confidence intervals of β, for each h from 5 to 10 weeks, are reported in Figure 2.

A limit of this approach is that it does not control for cohort effects (2020 vs 2018-19). As a more robust alternative a Regression Difference in discontinuity design (DRD) estimator can be proposed with the following equation:

Log[P(Y_it_=1)/P(Y_it_=0)] =

α+βD_it_ +δCohort_it_ + θD_it_Cohort_it_ + γ_1_(X_it_ - c)+ γ_2_D_it_(X_it_ - c)+γ_3_(X_it_ - c)Cohort_it_ + γ_4_D_it_(X_it_ - c)Cohort_it_

where Cohort_it_ = 1 for year 2020 (study cohort/period), Cohort_it_ = 0 for 2019-18 (control cohort/period).

Once estimated coefficients, and exponentiated they can be interpreted as odds-ratios (OR). In particular:

exp(δ): odds of death in 2020/odds of death in 2018-19, during the period before lockdown (OR_1_);

exp(β+θ): odds of death in 2020/odds of death in 2018-19, in the period after the lockdown (OR_2_);

exp(θ)= OR_2_/OR_1_: odds ratio (risk of death among post and pre lockdown) in 2020/odds ratio (risk of death among post and pre lockdown) in 2019-18, thus the treatment/lockdown effect in the study period controlling for cohort effect.

This DRD was estimated within several time-windows (from 5 to 10 weeks) around the lockdown date (March 9^th^). Point estimates and confidence intervals of θ, for each h from 5 to 10 weeks, are reported in Figure 3 and Figure A2.

Both models are estimated using Firth’s penalized maximum likelihood useful to manage problems of separation and rare-unbalanced events in situations of small sample sizes, that may occur in the shorter time windows. An alternative to Firth’s method is the exact method which, however, is known to be computationally intensive with more than one predictor or with a sample size exceeding 100 [41].
